# Physicochemical, Nutritional, and Antinutritional Properties of Pretreated Pulp and Whole Fruit Flours From a Plantain‐Like Hybrid (*CARBAP K74*) and a Plantain Landrace (*Batard*) at Different Post‐Harvest Ripening Stages

**DOI:** 10.1002/fsn3.71541

**Published:** 2026-02-19

**Authors:** Annie Takam Ngouno, Cédric Kendine Vepowo, Dallonnes Fangueng Kamgo, Samiha Boutaleb, Jean‐Luc Hornick, Marie‐Louise Scippo, Gérard Bertin Ngoh Newilah, Caroline Douny

**Affiliations:** ^1^ University of Liège Liège Belgium; ^2^ University of Dschang Dschang Cameroon; ^3^ Centre Africain de Recherches sur Bananiers et Plantains (CARBAP) Douala Cameroon; ^4^ University of Douala Douala Cameroon; ^5^ University of Yaoundé 1 Yaoundé Cameroon

**Keywords:** antinutritional factors, heat treatment, nutritional quality, plantain landrace, plantain‐like hybrid, whole fruit flour

## Abstract

Plantain flours offer a valuable preservation method with economic and nutritional benefits, especially for coeliac‐friendly foods, but their quality may decline if production techniques are not well controlled. This study is one of the first to comprehensively compare pulp and whole‐fruit flours across ripening stages, combining physicochemical, nutritional, and multivariate analyses. It specifically examines the effects of two heat treatments (blanching at 85°C for 5 min and precooking at 100°C for 15 min) on the quality of flours derived from the pulps and whole fruits of two plantain clones (*Batard* and *CARBAP K74*) at ripening stages 1, 3, and 5. Results revealed that variety, ripening stage, matrix, and treatment significantly influenced (*p* < 0.05) the quality of the flours. Treatments significantly affected the flour's color (L*, a*, b* parameters); blanching increased the browning index, whereas precooking decreased it. The flours exhibited acceptable moisture, pH, and soluble solids values, confirming good product stability. Carbohydrates were the predominant component (68%–85%), followed by fibers (1.9%–10.4%), proteins (2.5%–4.4%), ashes (1.7%–4%), and lipids (0.6%–2.1%). While the main fatty acids were palmitic, linoleic, and alpha‐linolenic acids. Antinutritional factors such as phytates, oxalates, and tannins were present at low levels. Multivariate analyses (PCA and HCA) revealed a clear separation of samples according to maturity stage, variety, and thermal treatment, grouping them into two main classes and identifying six superior flours from precooked whole *CARBAP K74* clone. These findings highlight their potential at ripening stages 3 and 5 for use in functional foods and as sustainable alternatives to wheat‐based products.

## Graphical abstract

This study evaluated pulp and whole‐fruit flours from *Batard* and *CARBAP K74* across ripening stages, showing that heat treatments, especially precooking, improved color, reduced browning, and lowered antinutritional factors. Multivariate analyses revealed strong influences of variety, maturity, and treatment on flour properties, with precooked whole‐fruit *CARBAP K74* at stages 3 and 5 showing the best nutritional qualities with higher fiber content. These flours demonstrate strong potential for infant porridges, bakery products, and sustainable wheat substitution in functional foods.

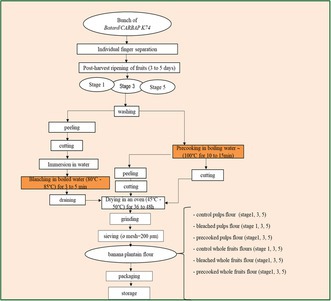

## 1 Introduction

Plantain (*Musa paradisiaca* AAB) is one of the most important staple foods for millions of people living in the tropics (Loranger‐Merciris et al. 2023) and plays a vital role in food security. Cameroon stands out as a key player in Africa, with production rising to 4.6 million tons, despite a slight decrease in cultivated areas (FAO 2024). Most of the plantains produced in the country are consumed locally, with only 5% dedicated to export (Falk 2018). Plantains are eaten by the entire population at various ripening stages (from stage 1 to 7), and cooking methods vary greatly depending on consumers' dietary preferences (Ngoh Newilah et al. 2024).

Despite its economic and nutritional importance, the plantain sector suffers from significant post‐harvest losses, estimated at around 40%. These losses are mainly due to poor transport conditions, inadequate marketing systems, and limited preservation techniques, resulting from the fruit's high perishability (Bancal and Ray 2024). One solution is the processing of plantains into high‐value derivatives, such as flour, which offer a longer shelf life and wide applications in human nutrition.

Developing countries that do not produce wheat are particularly vulnerable to food insecurity due to import constraints. With growing economic, health, and military crises (such as the COVID‐19 pandemic and the Russia‐Ukraine war) (von Braun et al. 2024). Furthermore, the wheat milling process removes the bran, which is rich in minerals and fibers, thereby reducing the nutritional quality of wheat flour. As a result, the FAO and various governments advocate for policies promoting the total or partial substitution of wheat in bakery products with locally produced flours (Fofiri and Temple 2023). Flour made from plant materials offers a preservation method with significant economic benefits. It can also be used to produce complementary foods that improve the nutritional status of people and those with wheat gluten intolerance (Luque et al. 2024). Plantain pulp flour is one of the primary derivatives obtained from processing plantains in Cameroon. Considering the challenges in peeling certain plantain varieties, the nutrients present in the peel, and the potential for valorising plantain peels, whole plantain fruit flours can be produced. Several studies, including that of Wani and Dhanya (2025), have shown that plantain peels contain many beneficial nutrients such as fibers, bioactive compounds, minerals, and vitamins. However, studies by Mundéné‐Timothée et al. (2024) have demonstrated that various technological processes (chemical and physical pretreatments) used during the transformation of plantain pulp into flour can affect the final quality of the flours. Several techniques can be implemented during the production of plantain flour to ensure acceptable quality. In developed countries, flour production utilizes sophisticated techniques such as controlled drying, milling optimization, and enzymatic wheat conditioning (Dziki 2023). In contrast, developing countries rely on improved traditional processing methods and simple, cost‐effective blanching techniques involving boiling water, pre‐cooking, or citric acid solutions (Ngoh et al. 2017). However, one major challenge during processing is controlling enzymatic browning, which causes discoloration of plantain slices (“*cossettes*”) and negatively affects the sensory and nutritional qualities of the resulting flour (Kiin‐Kabari 2020; Gupta et al. 2022). Given the need for efficient, low‐cost processing techniques and the potential of underutilized varieties, this study focuses on the plantain‐like hybrid *CARBAP K74*, a clone, a variety with excellent agronomic properties and resistance to some diseases such as Banana Streak Virus (eBSV) yet still underexploited compared to local cultivars such as *Batard*. This study investigates the impact of thermal treatments, specifically blanching and precooking, on the color, browning index, fatty acid profile, and physicochemical, nutritional, and anti‐nutritional properties of plantain flours produced at different ripening stages. It represents one of the first comprehensive assessments comparing pulp and whole‐fruit flours through combined physicochemical, nutritional, and multivariate analyses. The research aims to optimize processing conditions that enhance the nutritional and functional quality of plantain flours while meeting industrial requirements. Blanching and precooking are low‐cost, practical methods that effectively inactivate browning enzymes and reduce anti‐nutritional factors. Blanching helps retain color and heat‐sensitive nutrients, whereas precooking, being more intense, induces partial starch gelatinization that improves digestibility and overall flour quality. Furthermore, the comparison between pulp and whole‐fruit flours reflects a dual valorization approach that not only capitalizes on the nutritional richness of the peel particularly its fiber, compound contents but also reduces processing time and raw material waste.

## 2 Methodology

### 2.1 Materials and Samples

The plantain bunches were harvested at optimal physiological maturity from one of the African Research Centre on Bananas and Plantains (CARBAP) experimental plots located in Njombé (latitude: 4° 34′ 59.99″ N, longitude: 9° 39′ 59.99″ E, Littoral, region of Cameroon).

**FIGURE 1:**
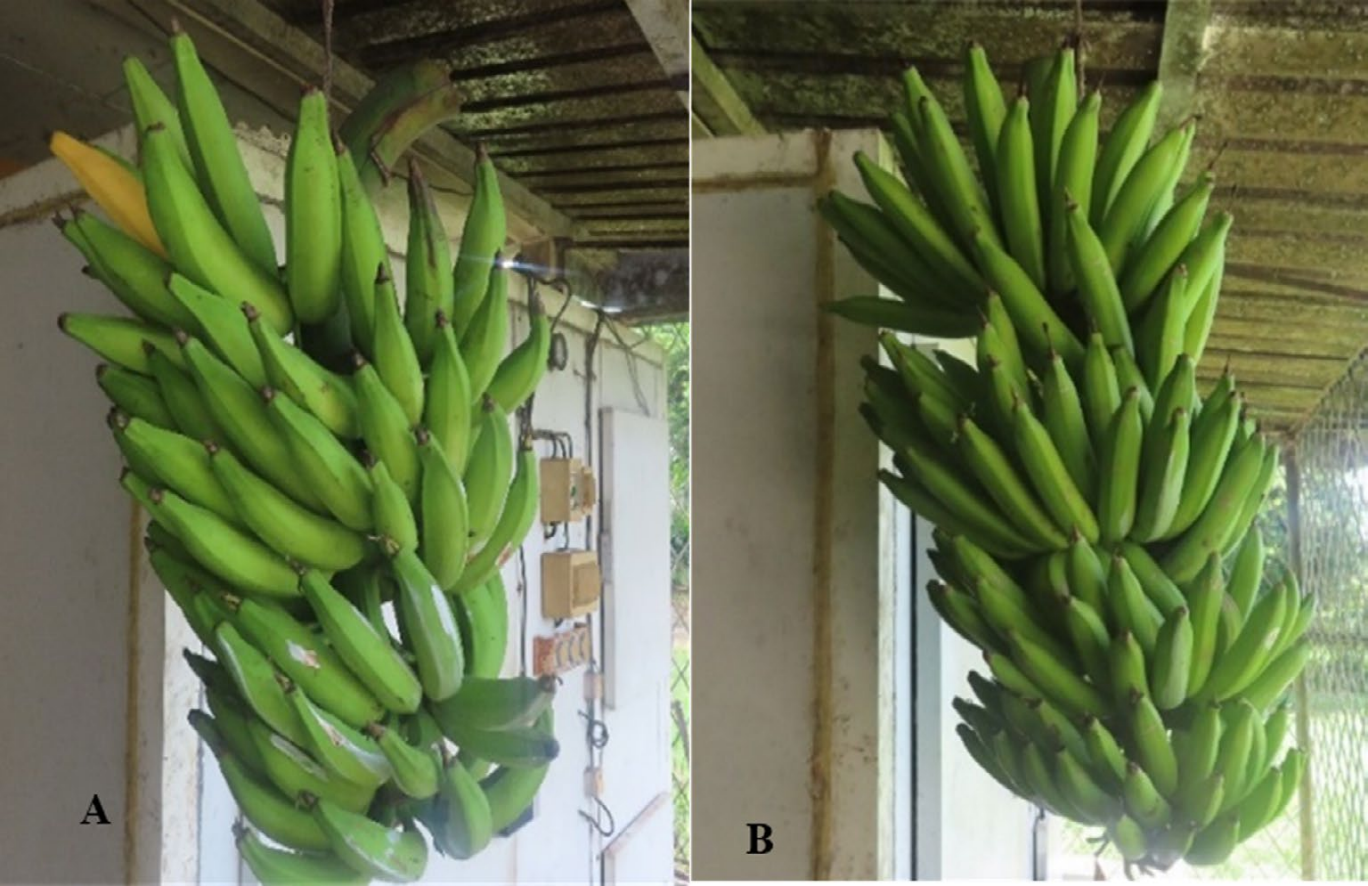
Bunch of *Batard cv*. (A) and *CARBAP K74* (B).

The plant material consisted of two plantain clones at three post‐harvest ripening stages (Figure 1). These included: a local plantain variety (*Batard*), and a plantain‐like (*CARBAP K74*). The choice of clones is based on a comparative approach intended to valorize the *CARBAP K74* hybrid, a variety still underexploited despite its resistance to some diseases such as Banana Streak Virus (eBSV) and pests and its excellent agronomic performance, compared with the landrace *Batard* variety, which is widely consumed in Central Africa, particularly in Cameroon. This comparison allows a better evaluation of the technological and nutritional potential of the hybrid in the processing of plantain into flour. At harvest, fruits from the bunches were selected, randomized and allowed to ripen at room temperature (27°C ± 2°C) in opened boxes.

**FIGURE 2:**
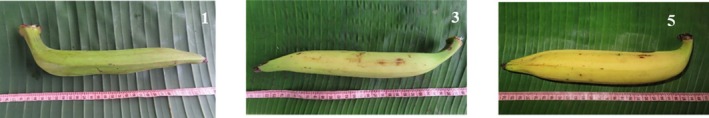
Ripening stages of plantain fruits (1 = unripe; 3 = start ripe; 5 = ripe).

Ripening stage of fruits, shown in Figure 2, were defined based on peel color changes, as recommended by Dadzie and Orchard (1997). Fruits at ripening stage 1 (unripe), ripening stage 3 (start ripe), and ripening stage 5 (ripe) exhibit peel colors of green, mixed green and yellow, and yellow with green tips, respectively. These 03 ripening Stages were selected to represent unripe, start ripe, and ripe fruits, allowing evaluation of the effects of fruit maturity on flour quality. At each stage of post‐harvest ripening, the plantain fruits were separated into six batches: (1) control whole fruit flour (pulp + peel), (2) control pulp flour, (3) blanched whole fruit flour, (4) blanched pulp flour, (5) precooked whole fruit flour, (6) precooked pulp flour.

### 2.2 Chemicals and Reagents

Concentrated sulfuric acid (H_2_SO_4_), potassium sulfate (K_2_SO_4_), copper sulfate (CuSO_4_), boric acid (H_3_BO_3_, 4.5%), methyl blue, methyl red, sodium hydroxide (NaOH), hydrochloric acid (HCl), ethylenediaminetetraacetic acid (EDTA), disodium hydrogen phosphate (Na_2_HPO_4_), sodium tetraborate decahydrate (Na_2_B_4_O_7_·10H_2_O), sodium dodecyl sulfate (SDS), triethylene glycol (antifoaming agent), thermostable alpha‐amylase, oxalic acid, potassium permanganate (KMnO_4_), and phytic acid were used in the experiments. All reagents were of analytical grade and obtained from Sigma‐Aldrich (St. Louis, MO, USA).

### 2.3 Flour Production

**FIGURE 3:**
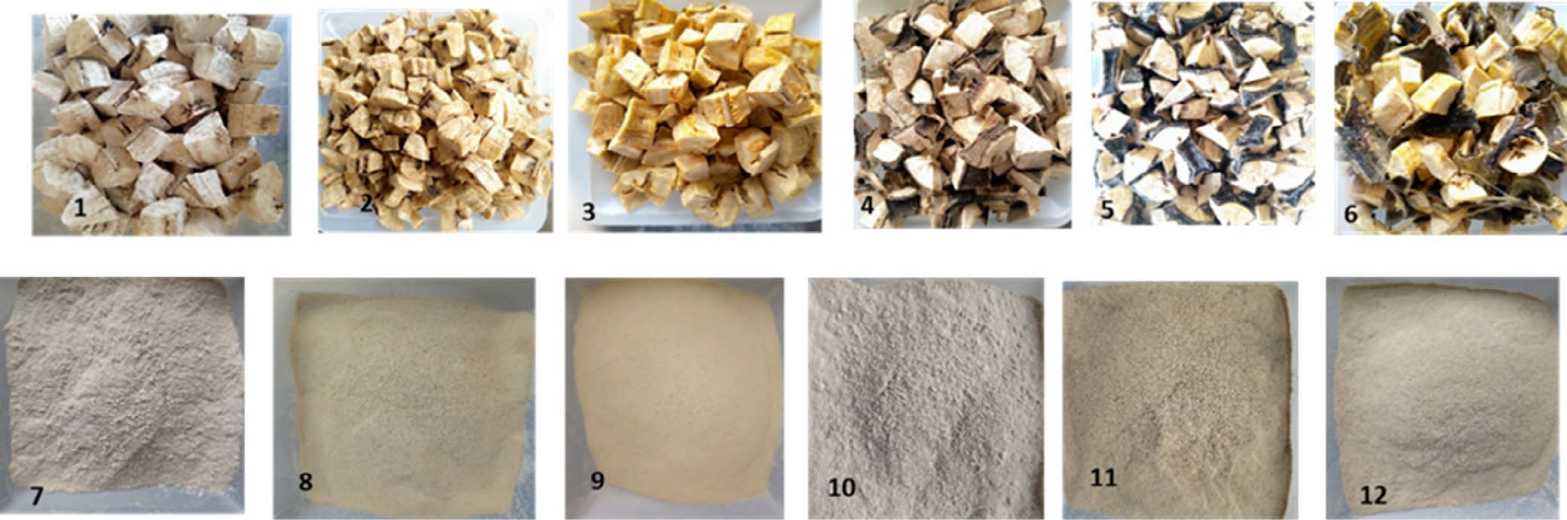
“Cossettes” and flours obtained from *Batard cv*. (1) Control pulp dried, (2) blanching pulp dried, (3) precooked pulp dried, (4) control whole fruit dried, (5) blanching whole fruit dried, (6) precooked whole fruit dried, (7) control pulp flour, (8) blanching pulp flour, (9) pre‐cooked pulp flour, (10) control whole fruit flour, (11) blanching whole fruit flour, and (12) pre‐cooked whole fruit flour.

**FIGURE 4:**
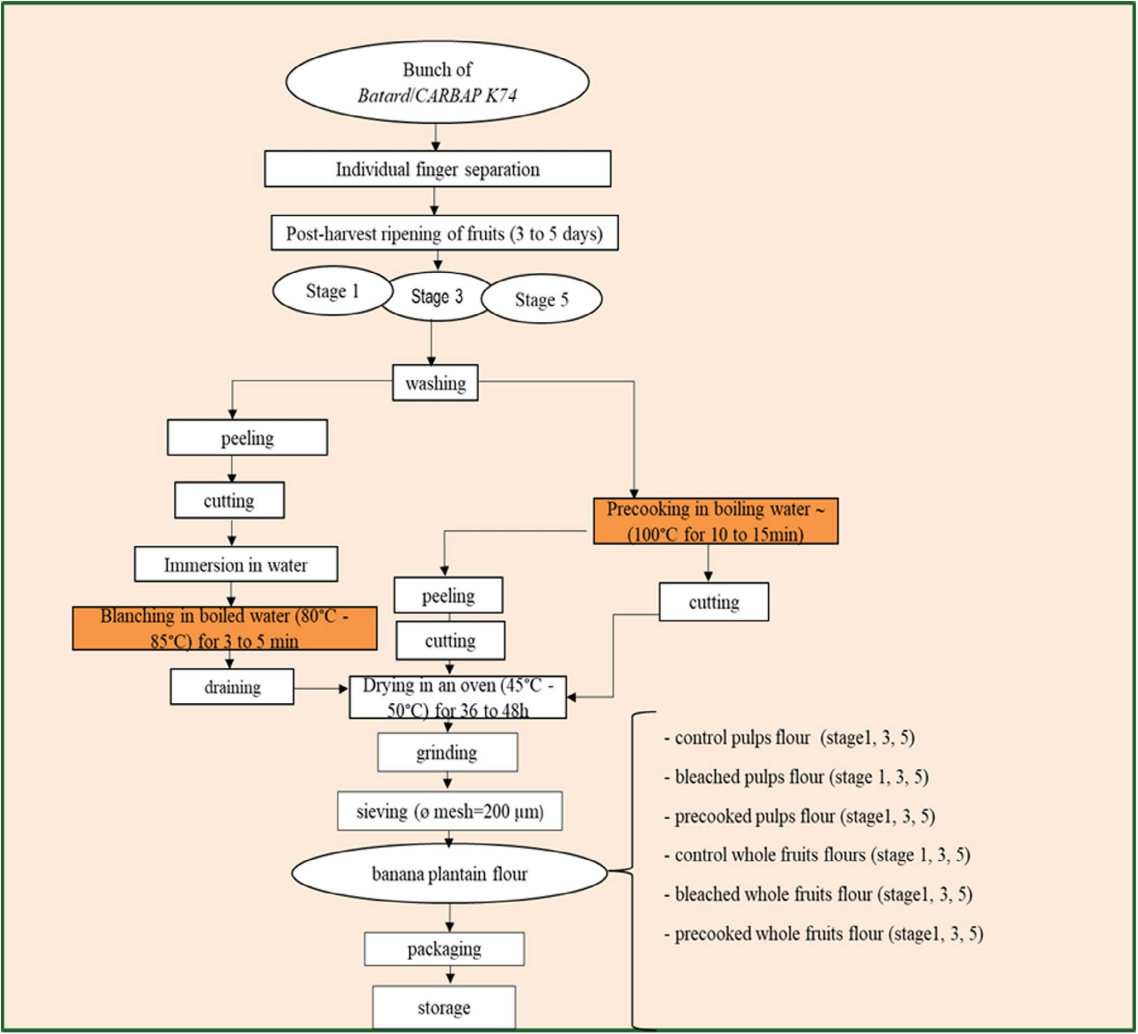
Flow chart of banana flour production.

Two heat treatments were used to produce flours: water blanching, which is a less costly technique, was carried out at 85°C for 5 min, and precooking, which is a technique similar to blanching but performed in boiling water for a longer duration, was carried out at 100°C for 15 min. Precooking was implemented on fruits with the peels (whole fruits), whereas blanching required the fruits (pulp or whole fruits depending on the kind of flour) to be sliced into 1 cm^3^. The objective of these two treatments (blanching and precooking) was to inactivate an enzyme called polyphenol oxidase, responsible for the blackening of plantain pulp exposed to oxygen (enzymatic browning), to reduce the bacterial load and to improve the anti‐nutritional quality of the flours. Precooking was specifically chosen because it is widely used in traditional banana‐based processing and partially gelatinizes starch, thereby enhancing digestibility. After treatment of the pulps and whole fruits, they were dried in a Memmert drying oven with hot air flow at temperatures ranging between 45°C and 50°C for 48 to 72 h depending on fruits postharvest maturation. The “*cossettes*” obtained were ground using an ordinary grinder (Retsch SM100, Germany) and the flours obtained were sifted using a stainless‐steel sieve with a mesh diameter of 200 μm. Finally, the flours obtained were preserved for subsequent analyses (Figure 3). The flour production process diagram is presented in Figure 4.

### 2.4 Physicochemical Analysis

The determination of water content (WC) was carried out according to the AOAC (1990) 925.10 method, based on measuring the weight loss of samples after drying at 105°C until the complete removal of free water and volatile substances.

Ash content was determined by burning around 5 g of a sample at 550°C for 24 h in a muffle furnace according to the AOAC 923.03 method (AOAC 2012). The ash and moisture content were both determined in triplicates.

The refractive index was determined using a handheld refractometer (REF 113, °Brix, 0–32 ATC), and the device was aligned toward a light source to read the refractive index (RI), which was then used to determine the soluble solids content (Dadzie and Orchard 1997).

The pH of the flours was measured using a non‐destructive method with a pH meter, a combined electrode, and a temperature probe, following ISO standard 2917:1999 (ISO, 1999).

Total titratable acidity was measured through titration using a 0.1 N sodium hydroxide (NaOH) solution until a persistent pink color was observed for approximately 10 s (Dadzie and Orchard 1997).

The determination of the true color in the SCE (Specular Component Excluded) mode of the flours was based on the evaluation of light reflected to the chromameter (Minolta CR‐400, Japan) coordinated by the L*, a*, b* parameters (Hunt 1991). L* represents lightness; a* represents red‐green; b* represents yellow‐blue. Browning evaluation was carried out following the method described by (Pathare et al. 2013) with:

$$\text{Browning index}\;\left(\mathrm{BI}\right)=\frac{100\;\left(x-0.31\right)}{0.17}\;\text{where}\;X=\frac{\left(a*+1.75L*\right)}{\left(5.645L*+a*-0.3012b*\right)}$$

### 2.5 Nutrients and Antinutrients Analysis

The crude protein content of the flour samples was determined by the Kjeldahl method according to (AOAC 2012). The protein content was calculated using the conversion factor of 6.25. This method involves three main steps: wet digestion, distillation with Bioblock Scientific, semi‐automatic distiller 1001, and titration. For the digestion step, 3 g of sample were placed in a boiling tube with 25 mL of concentrated sulfuric acid and one catalyst tablet (containing 5 g K_2_SO_4_, 0.15 g CuSO_4_, and 0.15 g TiO_2_). The mixture was heated at a low temperature until complete digestion was achieved. The digest was then diluted with 100 mL of distilled water, followed by the addition of 10 mL of 40% NaOH and 5 mL of Na_2_S_2_O_3_ (anti‐bumping agent). The liberated ammonia was collected into 10 mL of boric acid solution. Total protein was calculated by multiplying the nitrogen content by the conversion factor of 6.25. The protein content was then calculated using the following formula:

$$\begin{array}{c} \text{Proteins}\;\left(\%\right)=\frac{\left(\text{Volume}\;\mathrm{HCl}\;\text{sample}-\text{Volume}\;\mathrm{HCl}\;\text{blank}\right)\times N\times 1.401\times 6.25}{\text{sample weight}} \end{array}$$

where *N* = normality of HCl (0.1 N).

Total lipid content was determined according to the method of Folch et al. (1957), with slight modifications. Briefly, approximately 1 g of lyophilized sample was weighed, and in addition to 1 mL of water, the sample was then vortexed and let it stand for 10 min. After that, 40 mL of chloroform/methanol (2/1; v/v) were added into the test tubes, and they were placed on the rotary shaker for overnight agitation. The samples were then filtered into a 50 mL Falcon tube and filtered through a funnel containing paper filter and sodium sulfate. Furthermore, 8 mL of 0.88% potassium chloride was added, and samples were centrifuged at 3900 rpm for 10 min. Subsequently, the upper phase (methanol/water) of the extracts was removed with a water pump, and 10 mL of the chloroform phase extract was poured into a test tube for evaporation of the solvent in an oven at 60°C overnight. Finally, the dried tubes were weighed, and the total fat content was calculated. The result of fat content was expressed as grams of fat per 100 g of dried sample.

Determination of fibers content was assessed according to standard ISO 16472:2006 (ISO 2006) using a system, a neutral detergent (ND) solution and thermostable α‐amylase. FiberBags were dried at 105°C for 1 h, cooled in a desiccator, and weighed (value α). Approximately 0.5 g of the sample (value β), previously dried to determine its dry matter content, was placed in each FiberBag. The ND solution was prepared by dissolving NaOH, EDTA, Na_2_HPO_4_, and Na_2_B_4_O_7_·10H_2_O in water, followed by the addition of sodium lauryl sulfate and triethylene glycol as an antifoaming agent. The solution was adjusted to pH 7.0 ± 0.05 and made up to 1 L with distilled water. FiberBags containing the samples were immersed in 50 ± 5 mL of ND solution with boiling stones and an antifoam agent. The mixture was brought to a gentle boil, and 2 mL of thermostable α‐amylase solution was added. Samples were digested under gentle boiling for approximately 60 min, ensuring free movement of the FiberBags in the solution. After digestion, the FiberBags were removed, drained, and rinsed several times with hot water until no detergent residues remained. The obtained residues represented the neutral detergent fiber (NDF) fraction.

$$\%\mathrm{NDF}=e^{x}=\frac{\left(\left(\chi -\alpha \right)-\left(\delta -\zeta \right)\times 100\right)}{\beta }$$

Blank value: *ζ* = *δ*—*ψ*. *α* = Weight of the FiberBag (g), *β* = Weight of the sample (g), *χ* = Weight of the crucible and dried FiberBag (g), *δ* = Weight of the crucible and ash (g), and *ζ* = Blank value of the empty FiberBag (g).

The fatty acid profile was determined according to Douny et al. (2015) method by gas chromatography (GC) (Thermo Fisher Scientific, USA) using a CPSil88 column (100 m × 0.25 mm; 0.2 μm) (Varian, Agilent Technologies, Santa Clara, California, USA) and analyzed with a PolarisQ ion trap mass spectrometer (MS) (Thermo Fisher Scientific, USA).

The carbohydrate content was calculated using the following equation (Kim et al. 2007):

$$\text{Carbohydrate content}\%=100\%-\left(\text{moisture}+\text{proteins}+\mathrm{fat}+\mathrm{ash}\right)\%.$$

The oxalate content was determined using the modified titration method of Aina et al. (2012). In the presence of sulfuric acid and under heat, oxalic acid is oxidized by potassium permanganate. The oxidation of oxalic acid is marked by a color change to a persistent pink, lasting for a few seconds, which indicates the end of the reaction.

The determination of phytate content in the flours was performed using the method described by AOAC (1990). This method is based on the property of phytates to form stable and insoluble complexes with ferric ions in an acidic pH solution, where the source of phosphorus is phytic acid.

Total tannins were evaluated using the protocol described by Ndhlala et al. (2007). This method relies on the complexation of tannins with a ferric reagent in an acidic alcoholic medium, producing a red coloration measured at 550 nm using a spectrophotometer (Model 752G/752N). The intensity of the color is directly proportional to the tannin concentration in the sample.

### 2.6 Statistical Analysis

The collected data were stored in a database designed in Excel. Statistical analyses were carried out using SAS 9.4 and XLSTAT 2019. ANOVA followed by Tukey's post hoc test was performed for all measured parameters to determine significant differences among treatments, variety, ripening stage, and matrix. Statistical significance was considered at *p* < 0.05. Principal Component Analysis (PCA) and Hierarchical Cluster Analysis (HCA) were used to determine correlations between the variables and observations, and to identify different groups through a dendrogram, using XLSTAT software. All experiments were conducted in triplicate, and the results were presented as mean ± standard error of three replicates.

## 3 Results and Discussion

### 3.1 Proximate and Basic Physicochemical Properties of Flours

Tables 1 and 2 present the physicochemical parameters of plantain flours. ANOVA showed that Variety, thermal treatment, ripening stage, and matrix significantly influenced the physicochemical parameters of plantain flours, with each factor contributing differently to variations in water content, ash, pH, total soluble solids, and titratable acidity (Table 2).

**TABLE 1:** Physicochemical characteristics of control and treated flours obtained from unripe, start ripe, and ripe pulps and whole plantain fruits.

Ripening stage	Matrix	Treatments	*Batard*					*CARBAP K74*				
			WC (%)	Ash (%)	pH	TSS (°Brix)	TTA (meq/100 g)	WC (%)	Ash (%)	pH	TSS (°Brix)	TTA (meq/100 g)
Unripe (stage 1)	Pulps	Control	9.6 ± 0.2	2.15 ± 0.0	6.8 ± 0.1	5.2 ± 0.1	1288.9 ± 76	9.5 ± 0.4	2.44 ± 0.0	6.6 ± 0.2	9.2 ± 0.7	3644.4 ± 203
		Blanching	8.6 ± 0.2	1.68 ± 0.0	6.9 ± 0.4	4.0 ± 0.1	1022.2 ± 77	9.1 ± 0.1	2.02 ± 0.0	6.4 ± 0.1	4.8 ± 0.7	4355.6 ± 335
		Precooking	8.6 ± 0.3	2.34 ± 0.0	6.7 ± 0.5	5.2 ± 0.1	2088.9 ± 77	8.5 ± 0.3	2.55 ± 0.0	6.6 ± 0.2	6.4 ± 0.0	4000 ± 133
	Whole fruits	Control	8.3 ± 0.3	2.90 ± 0.0	6.8 ± 0.1	5.2 ± 0.1	2088.3 ± 133	8.9 ± 0.2	3.37 ± 0.0	6.9 ± 0.3	10.4 ± 0.7	4044.4 ± 204
		Blanching	8.3 ± 0.2	2.74 ± 0.0	7.0 ± 0.4	4.0 ± 0.0	1777.8 ± 77	7.8 ± 0.1	3.25 ± 0.2	6.9 ± 0.2	5.6 ± 0.7	4711.1 ± 439
		Precooking	8.3 ± 0.1	2.85 ± 0.0	6.7 ± 0.5	5.2 ± 0.0	2133.9 ± 77	7.9 ± 0.2	3.28 ± 0.1	6.7 ± 0.2	8.0 ± 0.7	5288.9 ± 278
Start ripe (stage 3)	Pulps	Control	8.1 ± 0.1	1.99 ± 0.1	6.0 ± 0.2	22.8 ± 0.7	2711.1 ± 77	10.5 ± 0.9	2.37 ± 0.1	6.4 ± 0.1	38 ± 0.8	5022.2 ± 336
		Blanching	7.2 ± 0.2	1.64 ± 0.1	5.7 ± 0.1	13.6 ± 0.0	2222.2 ± 77	8.8 ± 0.5	2.30 ± 0.0	5.8 ± 0.3	28 ± 0.8	5822.2 ± 335
		Precooking	7.3 ± 0.3	2.31 ± 0.0	5.8 ± 0.0	18.8 ± 0.7	2977.8 ± 77	8.8 ± 0.2	2.55 ± 0.0	6.0 ± 0.1	31.6 ± 1.0	4533.3 ± 133
	Whole fruits	Control	8 ± 0.0	2.94 ± 0.0	6.4 ± 0.1	22.8 ± 0.7	3777.8 ± 203	11.4 ± 0.2	3.55 ± 0.2	6.9 ± 0.1	37.2 ± 1.5	5066.7 ± 26
		Blanching	7.4 ± 0.1	2.58 ± 0.0	6.4 ± 0.1	13.2 ± 0.5	2977.8 ± 77	10.1 ± 0.2	3.33 ± 0.0	6.3 ± 0.2	33.2 ± 1.5	6755.1 ± 235
		Precooking	6.8 ± 0.1	2.59 ± 0.0	5.7 ± 0.3	22.0 ± 0.6	4533.3 ± 77	8.3 ± 0.9	3.78 ± 0.0	6.1 ± 0.3	30.4 ± 0.0	7200 ± 230
Ripe (stage 5)	Pulps	Control	9.5 ± 0.7	1.90 ± 0.0	6.0 ± 0.2	34.8 ± 0.7	3333.3 ± 133	11.8 ± 1.0	2.62 ± 0.0	6.1 ± 0.1	48.8 ± 1.0	5600 ± 133
		Blanching	9.3 ± 0.1	1.62 ± 0.0	5.7 ± 0.1	28.4 ± 0.7	3022.2 ± 77	9.5 ± 0.6	2.59 ± 0.1	5.7 ± 0.3	47.2 ± 0.0	6533 ± 133
		Precooking	8.5 ± 0.3	2.24 ± 0.0	5.9 ± 0.3	28.8 ± 0.7	3466.7 ± 300	9.3 ± 0.4	3.98 ± 0.0	6.2 ± 0.6	44.8 ± 1.0	7600 ± 133
	Whole fruits	Control	10.1 ± 0.1	2.67 ± 0.0	6.3 ± 0.2	34.4 ± 0.7	3911.1 ± 154	12.0 ± 0.3	3.76 ± 0.0	6.4 ± 0.1	49.2 ± 1.0	8088.9 ± 154
		Blanching	8.6 ± 0.2	2.73 ± 0.0	6.0 ± 0.1	28.4 ± 0.7	2222.2 ± 180	8.9 ± 0.2	3.78 ± 0.0	5.9 ± 0.3	43.6 ± 1.0	8622.2 ± 204
		Precooking	8 ± 0.0	2.67 ± 0.0	5.9 ± 0.1	30.4 ± 1.2	3066.7 ± 267	9.0 ± 0.2	3.58 ± 0.1	6.0 ± 0.6	41.2 ± 0.0	8577.8 ± 203

Abbreviations: pH, acidity index; TSS, total soluble solids; TTA, total titratable acidity; WC, water content.

**TABLE 2:** Mean squares from the analysis of variance (ANOVA) for the effects of variety, ripening stage, matrix, and treatment on the physicochemical properties of plantain flours.

Source of variation	WC	Ash	pH	TSS	TTA
Clone	10.67***	4.12***	0.04^ns^	10007***	235.75***
Ripening Stage	3.65***	0.15^ns^	1.91***	3173***	42.50***
Matrix	0.54^ns^	5.00***	0.44***	0.44^ns^	17.76***
Treatement	7.72***	0.42*	0.27***	90.17***	2.88^ns^

Abbreviations: pH, acidity index; TSS, total soluble solids; TTA, total titratable acidity; WC, water content; ns, not significant.

* *p* < 0.05.

*** *p* < 0.001.

Water content presented in Table 1 is an indicator of food's shelf life. High water content exposes food products to increased microbial spoilage and short shelf life (Ajayeoba et al. 2021). The moisture content of the flours decreases with the application of thermal treatments. Precooking shows an even greater reduction due to the loss of free water caused by membrane disruption, which accelerates dehydration during drying. The water contents of the flours were between 6.8 to 12.0 g/100 g DM, similar to those obtained on banana flour by Salazar et al. (2022) in Ecuador, which were within the range 9.9 and 11.3 g/100 g DM. Heat treatments reduce the moisture content of plantain flour. The water content values in the present work are in line with the quality limits of Codex Alimentarius (CODEX STAN 152‐1985) for flours, which are set at a maximum of 14% (CODEX 1985).

The ANOVA outcomes showed that the variety, treatments and matrix had a pronounced effect on ash content. The ash content of flours ranged from 1.6% to 4.0%. Blanching led to a decrease in ash content in both pulp and whole fruit flours at all ripening stages, whereas precooking caused an increase in pulp flours and a reduction in whole fruit flours. Ash content reflects mineral levels (calcium, magnesium, iron, potassium), essential for structural and regulatory functions. The flours' ash content (2.0–4.0 g/100 g) exceeded values reported by Campuzano et al. (2018) (1.6–2.6 g/100 g). Blanching caused mineral loss due to dissolution. Water‐soluble mineral components will be released during the blanching process. Setiarto et al. (2020) reported that boiling treatment reduced ash content from flour. Significant reduction occurs due to dissolving minerals into the immersion media which is accelerated by heating.

The pH of the control flours is significantly higher than that of the treated flours whatever the ripening stage and the matrix used. The pH (Table 1) of the different flours produced was between 5.56 and 7.04. These values are slightly higher than the values (5.0 to 6.2) obtained by Kumar et al. (2019). Regarding titratable acidity, precooking generally increases this parameter in whole fruit and pulp flours. The total titratable acidity of the flours varied between 1022.2 meq/100 g and 8622.2 meq/100 g. These values are greater than that obtained by Ngoh et al. (2017) which was 1741.2 meq/100 g. The decrease in pH and increase in total titratable acidity of flours made from plantains could be linked to the ripening stage and can be attributed to the activity of certain enzymes which during the maturation process, degrade the starch granules contained in fruits, leading to the formation and accumulation of soluble sugars (Singh et al. 2017).

The application of heat treatments (especially blanching) significantly reduces soluble solids content. During processing, these soluble solids compounds are leached out due to their water solubility. The amount of soluble solids determines the sugar content. It is generally expressed in °Brix with the relationship 20°Brix = 20% sugars in a medium. The soluble solids content (Table 1) of the different flours produced varied from 4° to 49° Brix. The values observed in this study are much higher than those obtained in Thailand by Donlao et al. (2020) which were in the range 0.1°–1.7° Brix. This difference is due to the ripening stage, the matrix, and the application of thermal treatments used in our study. These findings highlight the importance of considering both biological and technological factors in optimizing the quality attributes of plantain banana fruit flours.

Table 3 summarizes the effects of different sources of variation on color parameters (L*, a*, b*) and the browning index (BI). These parameters are used to assess visual changes in the flours, particularly those related to oxidation, thermal processing, or ripening. The browning index (BI) (Table 4) of flours ranged from 1.4 to 4.2 in *Batard* and from 2.6 to 10.2 in *CARBAP K74*. The BI was higher in whole fruit flours than in pulp flours for both cultivars due to peel, which is rich in phenolic compounds, precursors and enzymes for browning leading more pronounced browning reaction (Kaewjumpol et al. 2021). The BI, in our study are higher than 2.8 to 4.8 reported by Kaewjumpol et al. (2021) in their determination of the browning index of flours made from treated pulp. Precooking reduced the BI due to inactivation of PPO enzymes/browning enzymes. These findings are in line with those of Guessan et al. (2018), who noted that polyphenol oxidase activity, linked to browning, decreases above 45°C.

**TABLE 3:** Mean squares from the ANOVA for browning index (BI) and color parameters (L*, a*, b*) as affected by variety, ripening stage, matrix, and thermal treatment.

Source of variation	BI	L*	a*	b*
Clone	78.02***	808.45***	0.98**	1.1^ns^
Ripening stage	28/54***	69.86***	4.15***	25.12^ns^
Matrix	6.58**	427.11***	1.23***	2.1^ns^
Treatment	4.31**	103.25***	1.045***	2.49^ns^

Note: L*: Lightness, a*: Red‐Green component, b*: Yellow‐Blue component (*p* > 0.05).

Abbreviations: BI, Browning Index; ns, not significant.

** *p* < 0.01.

*** *p* < 0.001.

**TABLE 4:** Browning index and color parameters of control and treated flours obtained from unripe, start ripe and ripe pulps and whole plantain fruits (*Batard* and *CARBAP K74*).

Ripening stage	Matrix	Treatments	*Batard*				*CARBAP K74*			
			BI	L*	a*	b*	BI	L*	a*	b*
Unripe (stage 1)	Pulps	Control	1.4 ± 0.1	84.1 ± 0.1	0.33 ± 0.0	9.7 ± 0.1	2.6 ± 0.0	77.2 ± 0.1	0.24 ± 0.0	1.1 ± 0.3
		Blanching	2.4 ± 0.1	78.7 ± 0.6	0.57 ± 0.0	14.9 ± 0.2	3.5 ± 0.1	70.2 ± 0.4	0.16 ± 0.1	1.7 ± 0.2
		Precooking	1.4 ± 0.7	81.6 ± 1.0	−0.40 ± 0.1	14.6 ± 0.5	2.5 ± 0.1	75.1 ± 0.3	0.10 ± 0.1	0.6 ± 0.1
	Whole fruits	Control	1.9 ± 0.2	77.5 ± 1.0	0.69 ± 0.1	9.6 ± 0.2	4.2 ± 0.2	69.5 ± 0.9	0.71 ± 0.2	2.2 ± 0.3
		Blanching	2.4 ± 0.3	68.7 ± 0.4	0.61 ± 0.2	12.3 ± 1.0	5.7 ± 0.2	61.2 ± 1.0	0.45 ± 0.2	2.8 ± 0.1
		Precooking	1.4 ± 0.1	77.7 ± 0.9	0.58 ± 0.2	15.4 ± 0.3	3.4 ± 0.0	71.8 ± 0.1	0.34 ± 0.0	1.1 ± 0.2
Start ripe (stage 3)	Pulps	Control	1.4 ± 0.2	84.6 ± 0.2	0.04 ± 0.2	12.0 ± 1.0	5.5 ± 0.7	70.2 ± 1.2	0.42 ± 0.5	2.4 ± 1.0
		Blanching	2.5 ± 0.1	78.3 ± 0.3	0.29 ± 0.0	18.0 ± 0.3	4.6 ± 0.6	70.1 ± 1.0	0.31 ± 0.6	1.8 ± 1.0
		Precooking	1.5 ± 0.2	82.0 ± 0.0	−0.94 ± 0.1	19.5 ± 0.0	3.6 ± 0.2	74.5 ± 0.7	0.31 ± 0.1	0.7 ± 1.0
	Whole fruits	Control	2.3 ± 0.1	75.9 ± 0.9	0.73 ± 0.2	12.1 ± 0.4	6.9 ± 0.2	63.0 ± 1.1	0.81 ± 0.1	3.4 ± 1.0
		Blanching	3.5 ± 0.5	67.1 ± 0.3	1.27 ± 0.1	14.3 ± 0.2	5.9 ± 0.4	60.0 ± 0.7	0.73 ± 0.0	2.7 ± 0.6
		Precooking	2.0 ± 0.2	80.2 ± 1.4	−0.35 ± 0.2	18.8 ± 1.3	4.5 ± 0.3	69.7 ± 0.3	0.45 ± 0.1	1.4 ± 0.1
Ripe (stage 5)	Pulps	Control	3.1 ± 0.3	79.7 ± 1.8	0.92 ± 0.3	17.9 ± 1.2	8.6 ± 0.1	64.5 ± 0.1	1.88 ± 0.1	3.9 ± 0.2
		Blanching	4.2 ± 0.2	77.5 ± 0.7	1.51 ± 0.2	21.7 ± 0.3	7.1 ± 0.5	69.6 ± 1.0	1.83 ± 0.1	2.7 ± 0.0
		Precooking	3.1 ± 0.1	79.6 ± 0.1	−0.13 ± 0.0	25.2 ± 0.1	6.3 ± 0.2	67.3 ± 0.3	1.67 ± 0.3	3.3 ± 1.3
	Whole fruits	Control	4.0 ± 0.2	69.9 ± 0.4	1.7 ± 0.2	15.5 ± 1.1	10.2 ± 0.1	55.5 ± 1.2	1.8 ± 0.2	4.8 ± 0.5
		Blanching	4.1 ± 0.1	68.0 ± 0.6	1.45 ± 0.1	17.7 ± 0.1	7.6 ± 0.2	62.5 ± 1.8	1.24 ± 0.1	2.6 ± 0.6
		Precooking	3.9 ± 0.2	77.0 ± 0.4	0.61 ± 0.3	25.8 ± 0.2	6.8 ± 0.3	65.6 ± 1.0	1.97 ± 0.0	2.9 ± 1.0

Color represents a major aspect of the marketability of food. The Lab* parameter refers to a color measurement system called the CIELAB system (or Lab color space), commonly used in food science and other industries. Each parameter represents the following: L* (lightness) indicates the brightness of the color, ranging from 0 (black) to 100 (white), a* represents the hue on the green‐red axis, with negative values indicating a shift toward green and positive values toward red, b* represents the hue on the blue‐yellow axis, with negative values indicating a shift toward blue and positive values toward yellow.

L* parameter decrease and those of the a* and b* parameters increase with browning. Likewise, the values of the parameters a* decrease with the ripening and the effectiveness of the treatment applied. The L* parameter, which represents clarity, had values between 60.0 and 85.0. The values obtained (Table 4) in this study are lower than the values 95.9–98.59 obtained by Campuzano et al. (2018). The parameter a*, which corresponds to redness index, presented values between −0.94 and 1.97 during this study. Our values are in the same range with those obtained by Udomkun et al. (2021) who had −1.1 to 2.7. The parameter b* of the different flours produced had values ranging from 1.1 to 25.8 these values are quite similar to the values of 26.3–29.2 obtained by Virulchatapan and Luansakum (2020).

### 3.2 Nutrient Content of Flour

The nutritional parameters are summarized in Table 5, while the mean square values showing the effects of cultivar, matrix, and treatment on the proximate composition are presented in Table 6.

**TABLE 5:** **N**utritional parameters of control and treated flours obtained from unripe, start ripe and ripe pulps and whole plantain fruits (*Batard* and *CARBAP K74*).

Ripening stage	Matrix	Treatments	*Batard*				*CARBAP K74*			
			NDF (%)	Lipids (%)	Proteins (%)	Carbohydrates (%)	NDF (%)	Lipids (%)	Proteins (%)	Carbohydrates (%)
Unripe (stage 1)	Pulps	Control	1.95 ± 0.2	0.61 ± 0.1	2.71 ± 0.2	83.0 ± 0.2	3.81 ± 0.2	0.75 ± 0.1	4.02 ± 0.1	79.4 ± 0.3
		Blanching	3.84 ± 0.2	0.58 ± 0.0	2.51 ± 0.1	82.8 ± 0.7	5.01 ± 0.2	0.82 ± 0.2	3.66 ± 0.2	79.4 ± 0.7
		Precooking	5.00 ± 0.2	0.57 ± 0.0	2.60 ± 0.3	80.9 ± 0.3	5.47 ± 0.4	0.70 ± 0.1	3.85 ± 0.1	79.0 ± 0.4
	Whole fruits	Control	3.94 ± 0.4	0.81 ± 0.0	3.22 ± 0.0	80.8 ± 1.0	6.76 ± 0.4	1.80 ± 0.2	4.02 ± 0.1	75.2 ± 1.0
		Blanching	5.44 ± 0.5	0.83 ± 0.1	3.02 ± 0.0	79.6 ± 0.2	9.20 ± 1.0	1.57 ± 0.1	3.88 ± 0.2	74.3 ± 0.2
		Precooking	6.25 ± 0.3	0.92 ± 0.1	3.04 ± 0.0	78.7 ± 1.1	6.73 ± 0.3	1.58 ± 0.1	3.96 ± 0.1	76.5 ± 1.0
Start ripe (stage 3)	Pulps	Control	1.94 ± 0.1	0.69 ± 0.0	2.73 ± 0.0	84.6 ± 1.1	4.55 ± 0.4	0.91 ± 0.1	3.92 ± 0.2	77.8 ± 1.1
		Blanching	5.05 ± 0.0	0.64 ± 0.2	2.61 ± 0.2	82.9 ± 1.1	7.40 ± 0.7	0.74 ± 0.1	3.85 ± 0.1	76.9 ± 0.2
		Precooking	3.93 ± 0.0	0.63 ± 0.2	2.75 ± 0.1	83.1 ± 0.1	5.25 ± 0.2	1.01 ± 0.1	3.85 ± 0.3	78.6 ± 0.6
	Whole fruits	Control	4.79 ± 0.1	1.05 ± 0.1	3.21 ± 0.0	80.1 ± 0.7	5.23 ± 0.2	2.01 ± 0.3	3.94 ± 0.1	73.9 ± 0.3
		Blanching	6.53 ± 0.5	1.10 ± 0.1	3.18 ± 0.1	79.2 ± 1.3	8.00 ± 0.8	2.04 ± 0.1	4.17 ± 0.1	72.4 ± 0.2
		Precooking	5.50 ± 0.0	1.09 ± 0.1	3.21 ± 0.2	80.7 ± 0.4	6.10 ± 0.2	1.74 ± 0.2	4.06 ± 0.2	76.0 ± 0.4
Ripe (stage 5)	Pulps	Control	3.58 ± 0.4	0.81 ± 0.0	2.58 ± 0.0	81.6 ± 0.6	6.54 ± 0.3	1.10 ± 0.1	4.09 ± 0.1	73.8 ± 0.5
		Blanching	4.25 ± 0.3	0.74 ± 0.1	2.74 ± 0.0	81.4 ± 1.2	7.68 ± 0.4	0.78 ± 0.2	4.13 ± 0.3	75.3 ± 0.3
		Precooking	3.33 ± 0.1	0.64 ± 01	2.77 ± 0.0	82.5 ± 0.8	7.72 ± 0.3	0.75 ± 0.1	3.99 ± 0.1	74.3 ± 0.1
	Whole fruits	Control	2.84 ± 0.4	1.14 ± 0.0	3.06 ± 0.0	80.2 ± 0.4	9.86 ± 0.2	2.16 ± 0.3	4.36 ± 0.4	67.9 ± 0.5
		Blanching	5.48 ± 0.1	1.34 ± 0.2	3.28 ± 0.2	78.6 ± 0.5	10.34 ± 0.2	2.16 ± 0.1	4.09 ± 0.1	70.7 ± 0.4
		Precooking	3.42 ± 0.0	1.10 ± 0.1	3.16 ± 0.1	81.7 ± 0.6	9.72 ± 0.2	2.08 ± 0.1	4.10 ± 0.1	71.6 ± 0.7

Abbreviation: NDF, neutral detergent fibers.

**TABLE 6:** Mean squares of the effects of variety, ripening stage, matrix, and heat treatment on the proximate composition of plantain flours.

Source of variation	NDF	Lipids	Proteins	Carbohydrates
Clone	57.70***	2.459***	10.62***	332.75***
Ripening stage	4.73**	0.227***	0.72**	22.56***
Matrix	9.23**	4.73***	0.87***	97.51***
Treatment	9.14**	0.022^ns^	0.012^ns^	2.1^ns^

Abbreviations: NDF, neutral detergent fibers; ns, not significant.

** *p* < 0.01.

*** *p* < 0.001.

The content of NDF in a sample estimates the amount of cellulose, hemicellulose, and lignin present. The fiber is significantly influenced by heat treatments, matrix, and ripening stage. Fiber content ranged from 1.9% to 6.3% in *Batard* flours and 3.8% to 10.3% in *CARBAP K74* flours. Blanching and precooking increased fiber levels, with precooking being more effective at ripening stage 1 and blanching at stages 3 and 5 for both varieties. Dietary fibers are crucial for digestive health, weight management, and reducing cardiac and gastrointestinal risks (Fidrianny et al. 2018). Flours produced from the whole fruit exhibit higher fiber contents due to the contribution of the peels, which are rich in structural compounds such as cellulose, hemicellulose, and lignin (Jimenez Moreno et al. 2025). The fiber content (1.9–10.3 g/100 g DM) exceeded values reported by Soto‐Maldonado et al. (2020) for banana flours (3.9 g/100 g DM) likely.

Lipid content increased with ripening stage and matrix. Whole fruit flours have higher levels than pulp flours. Lipids content ranged from 0.6% to 2.2% on the flours. Heat treatments, especially precooking reduced lipid content at all ripening stages. Lipids provide 35%–40% of daily energy intake. In this study, lipid content obtained (0.6–2.2 g/100 g DM) was lower than obtained by Fadimu and Oladimeji (2018) (2.2–2.6 g/100 g DM). Variations may be due to plantain variety, and ripening stage.

Protein content ranged from 2.5% to 3.3% in *Batard* flours and 3.9% to 4.4% in *CARBAP K74* flours. Heat treatments reduced protein levels, with blanching causing the greatest reduction in both varieties. Proteins are essential nutrients. alongside carbohydrates, lipids, and vitamins. In this study, protein content (2.5–4.4 g/100 g DM) was slightly higher than values reported by Salazar et al. (2022) (3.4–3.7 g/100 g DM). Higher protein levels in whole fruit flours observed may result from amino acids like leucine, valine, and threonine in the peel (Blasco and Montano 2015).

ANOVA shows that heat treatments and ripening stage significantly affect carbohydrate in the flours. Carbohydrate content in flours varied slightly with ripening, ranging from 80.7% to 84.6% in *Batard* flours and 67.9% to 79.4% in *CARBAP K74* flours. Heat treatments reduce carbohydrate content by precooking, causing a greater loss than blanching. The higher temperatures and times in precooking cause a more significant breakdown of larger carbohydrate molecules into smaller, more easily dissolved ones, increasing the potential for loss. Carbohydrates are the primary energy source for the body, particularly for the brain, which relies solely on glucose (Arshad et al. 2025). The highest carbohydrate content was 84.6 g/100 g DM, lower than the 86.7% reported by Iliyasu and Ayo‐omogie Iliyasu et al. (2019) for banana flour.

Fatty acid profiles of *Batard* and *CARBAP K74* flours are presented on Tables 7 and 8. Heat treatments reduced palmitic and stearic acids but increased alpha‐linolenic and linoleic acids. Palmitic acid was the most abundant, followed by linoleic, alpha‐linolenic, stearic, and palmitoleic acids. Heat treatments lead to a decrease in saturated (∑SFA), monounsaturated (∑MUFA) fatty acids, and the *n*−6/*n*−3 ratio, while increasing polyunsaturated fatty acids (∑PUFA), including *n*−6 and *n*−3, regardless of whether the flour is made from pulp or whole fruit. Notably, the *n*−6/*n*−3 ratio decreases as ripening progresses. The lipid profile includes both saturated and unsaturated fatty acids, particularly essential ones such as linoleic acid (C18:2 *n*−6) and alpha‐linolenic acid (C18:3 *n*−3). The *n*−6/*n*−3 ratio (1.51–3.15) aligns with European Food Safety Authority (EFSA) recommended range (1%–4%). Unsaturated fatty acids are beneficial for reducing blood cholesterol, and *n*−3 fatty acids play a crucial role in preventing cardiovascular diseases (Takahashi et al. 2017), which are the second leading cause of death in France (DREES 2017).

**TABLE 7:** Fatty acid profile of control and treated flours obtained from unripe, start ripe and ripe pulps and whole plantain fruits.

Clone			*Batard*								CARBAP K74						
Fatty acids (g/100 g of flour)			Ac. saturated fat		Monounsaturated fatty acids			N‐3 polyunsaturated fatty acids	*n*−6 polyunsaturated fatty acids		Saturated fatty acids		Monounsaturated fatty acids			Ac. *n*−3 polyunsaturated fat	Ac. *n*−6 polyunsaturated fat
Ripening stage	Matrix	Treatments	C16:0	C18:0	C16:1 *n*−7	C18:1 *n*−9	C18:1 *n*−7	C18:3 *n*−3	C18:2 *n*−6	C20:4 *n*−6	C16:0	C18:0	C16:1 *n*−7	C18:1 *n*−9	C18:1*n*−7	C18:3 *n*−3	C18:2 *n*−6
Unripe (stage 1)	Pulps	Control	0.28	0.03	0.02	<LOQ	0.04	0.05	0.18	<LOQ	0.30	0.06	0.02	<LOQ	0.04	0.09	0.24
		Blanching	0.17	0.02	0.02	<LOQ	0.02	0.10	0.24	<LOQ	0.25	0.05	0.01	<LOQ	0.03	0.15	0.32
		Precooking	0.18	0.02	0.01	<LOQ	0.03	0.10	0.24	<LOQ	0.21	0.04	0.01	<LOQ	0.03	0.13	0.28
	Whole fruits	Control	0.36	0.04	0.02	<LOQ	0.04	0.10	0.24	<LOQ	0.97	0.18	<LOQ	<LOQ	0.06	0.15	0.46
		Blanching	0.27	0.03	0.01	0.03	0.03	0.15	0.31	<LOQ	0.64	0.13	0.01	<LOQ	0.05	0.21	0.53
		Precooking	0.32	0.03	0.02	0.03	0.03	0.15	0.33	<LOQ	0.68	0.13	0.01	<LOQ	0.05	0.19	0.51
Start ripe (stage 3)	Pulps	Control	0.24	0.03	0.01	<LOQ	0.03	0.13	0.23	<LOQ	0.27	0.05	0.01	<LOQ	0.04	0.18	0.35
		Blanching	0.18	0.03	0.01	<LOQ	0.03	0.14	0.24	<LOQ	0.21	0.04	0.01	<LOQ	0.03	0.15	0.30
		Precooking	0.17	0.03	0.01	<LOQ	0.03	0.15	0.23	<LOQ	0.30	0.07	0.01	<LOQ	0.04	0.19	0.37
	Whole fruits	Control	0.37	0.03	0.01	<LOQ	0.04	0.21	0.36	<LOQ	0.83	0.16	<LOQ	0.1	0.06	0.27	0.62
		Blanching	0.38	0.03	0.01	0.04	0.04	0.21	0.37	0.00	0.89	0.18	<LOQ	0.1	0.05	0.24	0.61
		Precooking	0.38	0.03	0.01	0.04	0.04	0.20	0.37	0.02	0.72	0.15	<LOQ	<LOQ	0.05	0.24	0.57
Ripe (stage 5)	Pulps	Control	0.25	0.02	0.01	<LOQ	0.04	0.23	0.26	<LOQ	0.35	0.03	0.01	<LOQ	0.04	0.27	0.40
		Blanching	0.21	0.02	0.01	<LOQ	0.03	0.21	0.25	<LOQ	0.23	0.02	0.01	<LOQ	0.03	0.19	0.29
		Precooking	0.18	0.02	0.01	<LOQ	0.03	0.17	0.22	<LOQ	0.23	0.03	0.01	<LOQ	0.03	0.18	0.27
	Whole fruits	Control	0.39	0.02	0.01	<LOQ	0.05	0.28	0.38	<LOQ	0.99	0.09	<LOQ	<LOQ	0.05	0.35	0.67
		Blanching	0.49	0.02	0.01	<LOQ	0.05	0.34	0.43	<LOQ	1.01	0.09	<LOQ	<LOQ	0.06	0.33	0.68
		Precooking	0.40	0.06	0.01	<LOQ	0.04	0.24	0.33	<LOQ	0.97	0.09	<LOQ	<LOQ	0.05	0.31	0.65

*Note:* C16:0: palmitic acid; C18:0: stearic acid; C16:1 *n*−7: palmitoleic acid; C18:1 *n*−9: oleic acid; C18:1 *n*−7: cis‐vaccenic acid; C18:3 *n*−3: alpha‐linolenic acid; C18:2 *n*−6: linoleic acid; C20:4 *n*−6: arachidonic acid; <LOQ: lower than the limit of quantification.

**TABLE 8:** Sum of fatty acids in control and treated flours obtained from unripe, start ripe, and ripe pulps and whole plantain fruits (*Batard* and *CARBAP K74*).

Clone				Batard					*CARBAP K74*					
Ripening stage	Matrix	Treatements	∑ SFA	∑MUFA	∑PUFA	∑ *n*−6	∑ *n*−3	n−6n−3	∑ SFA	∑MUFA	∑PUFA	∑ *n*−6	∑ *n*−3	n−6n−3
Unripe (stage 1)	Pulps	Control	0.32	0.06	0.23	0.19	0.05	3.31	0.37	0.05	0.33	0.24	0.09	2.65
		Blanching	0.19	0.04	0.34	0.24	0.10	2.32	0.3	0.05	0.47	0.32	0.15	2.13
		Precooking	0.19	0.04	0.33	0.24	0.10	2.50	0.25	0.04	0.41	0.28	0.13	2.13
	Whole fruits	Control	0.42	0.05	0.34	0.24	0.10	2.46	1.15	0.06	0.60	0.46	0.15	3.15
		Blanching	0.3	0.07	0.46	0.31	0.15	2.13	0.77	0.06	0.74	0.53	0.21	2.52
		Precooking	0.36	0.08	0.48	0.33	0.15	2.27	0.81	0.07	0.70	0.51	0.19	2.67
Start ripe (stage 3)	Pulps	Control	0.28	0.05	0.36	0.23	0.13	1.81	0.33	0.05	0.53	0.35	0.18	1.93
		Blanching	0.21	0.04	0.39	0.24	0.14	1.69	0.25	0.04	0.45	0.30	0.15	1.95
		Precooking	0.21	0.04	0.38	0.23	0.15	1.54	0.37	0.05	0.56	0.37	0.19	1.96
	Whole fruits	Control	0.42	0.06	0.57	0.36	0.21	1.73	0.99	0.12	0.89	0.62	0.27	2.32
		Blanching	0.42	0.09	0.59	0.37	0.21	1.74	1.06	0.12	0.85	0.61	0.24	2.52
		Precooking	0.41	0.10	0.58	0.38	0.20	1.93	0.87	0.05	0.81	0.57	0.24	2.37
Ripe (stage 5)	Pulps	Control	0.28	0.05	0.48	0.26	0.23	1.15	0.38	0.06	0.67	0.40	0.27	1.46
		Blanching	0.24	0.04	0.46	0.25	0.21	1.19	0.25	0.04	0.49	0.29	0.19	1.52
		Precooking	0.20	0.04	0.40	0.22	0.17	1.28	0.27	0.04	0.44	0.27	0.18	1.51
	Whole fruits	Control	0.42	0.06	0.67	0.38	0.28	1.34	1.08	0.05	1.02	0.67	0.35	1.91
		Blanching	0.52	0.06	0.76	0.43	0.34	1.27	1.10	0.06	1.01	0.68	0.33	2.05
		Precooking	0.47	0.05	0.58	0.33	0.24	1.38	1.06	0.05	0.96	0.65	0.31	2.06

*Note:* ∑ SFA: Sum of saturated fatty acids, ∑ MUFA: Sum of monounsaturated fatty acids, ∑ PUFA: Sum of polyunsaturated fatty acids, ∑ *n*−6: Sum of *n*−6 fatty acids, ∑ *n*−3: Sum of *n*−3 fatty acids, *n*−6/*n*−3 – Ratio of *n*−6 to *n*−3 fatty acids.

### 3.3 Antinutrients Content

The antinutrients content of flours is presented in Table 9, while the mean square values from the analysis of variance for antinutritional factors are provided in Table 10.

**TABLE 9:** Physicochemical parameters of control and treated flours obtained from unripe, start ripe, and ripe pulps and whole plantain fruits (*Batard* and *CARBAP K74*).

Ripening stage	Matrix	Treatments	*Batard*			*CARBAP K74*		
			Phytates (%)	Oxalates (%)	Tannins (%)	Phytates (%)	Oxalates (%)	Tannins (%)
Unripe (stage 1)	Pulps	Control	0.16 ± 0.0	0.07 ± 0.0	0.26 ± 0.0	0.12 ± 0.0	0.12 ± 0.0	0.12 ± 0.0
		Blanching	0.15 ± 0.0	0.08 ± 0.0	0.50 ± 0.1	0.11 ± 0.0	0.11 ± 0.0	0.11 ± 0.0
		Precooking	0.14 ± 0.0	0.17 ± 0.0	0.84 ± 0.1	0.09 ± 0.0	0.09 ± 0.0	0.07 ± 0.0
	Whole fruits	Control	0.20 ± 0.0	0.07 ± 0.0	0.55 ± 0.1	0.18 ± 0.0	0.18 ± 0.0	0.12 ± 0.0
		Blanching	0.14 ± 0.0	0.10 ± 0.0	0.76 ± 0.1	0.15 ± 0.0	0.15 ± 0.0	0.16 ± 0.0
		Precooking	0.14 ± 0.0	0.14 ± 0.0	0.97 ± 0.1	0.14 ± 0.0	0.14 ± 0.0	0.10 ± 0.0
Start ripe (stage 3)	Pulps	Control	0.09 ± 0.0	0.30 ± 0.0	0.31 ± 0.0	0.13 ± 0.0	0.13 ± 0.0	0.34 ± 0.0
		Blanching	0.08 ± 0.0	0.19 ± 0.0	0.15 ± 0.0	0.12 ± 0.0	0.12 ± 0.0	0.30 ± 0.0
		Precooking	0.08 ± 0.0	0.31 ± 0.0	0.29 ± 0.0	0.11 ± 0.0	0.13 ± 0.0	0.26 ± 0.0
	Whole fruits	Control	0.13 ± 0.0	0.27 ± 0.0	0.50 ± 0.0	0.24 ± 0.0	0.24 ± 0.0	0.39 ± 0.0
		Blanching	0.12 ± 0.0	0.22 ± 0.0	0.31 ± 0.0	0.19 ± 0.0	0.19 ± 0.0	0.34 ± 0.0
		Precooking	0.11 ± 0.0	0.26 ± 0.0	0.50 ± 0.1	0.17 ± 0.0	0.17 ± 0.0	0.31 ± 0.0
Ripe (stage 5)	Pulps	Control	0.09 ± 0.0	0.31 ± 0.0	0.37 ± 0.0	0.18 ± 0.0	0.18 ± 0.0	0.29 ± 0.0
		Blanching	0.14 ± 0.0	0.31 ± 0.0	0.16 ± 0.0	0.13 ± 0.0	0.13 ± 0.0	0.29 ± 0.0
		Precooking	0.20 ± 0.0	0.27 ± 0.0	0.45 ± 0.0	0.10 ± 0.0	0.11 ± 0.0	0.25 ± 0.0
	Whole fruits	Control	0.14 ± 0.0	0.29 ± 0.1	0.97 ± 0.1	0.15 ± 0.0	0.15 ± 0.0	0.27 ± 0.0
		Blanching	0.14 ± 0.0	0.20 ± 0.0	0.39 ± 0.1	0.19 ± 0.0	0.19 ± 0.0	0.26 ± 0.0
		Precooking	0.41 ± 0.0	0.18 ± 0.0	0.47 ± 0.1	0.14 ± 0.0	0.14 ± 0.0	0.26 ± 0.0

**TABLE 10:** Analysis of variance (mean squares) for antinutritional factors in plantain flours.

Source of variation	Phytates	Oxalates	Tannins
Clone	0.00^ns^	0.031***	0.565***
Ripening stage	0.004^ns^	0.032***	0.007^ns^
Matrix	0.02^ns^	0.000^ns^	0.14^ns^
Treatment	0.00^ns^	0.00 ^ns^	0.024^ns^

Abbreviation: ns, not significant.

*** *p* < 0.001.

The phytate contents (Table 9) of the various flours decreased with the heat treatments applied, with precooking leading to the greatest reduction. However, an exception was observed at ripening stage 5 for the *Batard* clone, where precooking tended to increase the phytate content. The phytate content in the flours ranged from 0.09% to 0.4%. These values are greater than those obtained by Akinjayeju et al. (2020) (0.03 g/100 g DM). The reduction in treated flours is due to the thermolabile nature of phytic acid by diffusion or hydrolysis through phytase activation at elevated temperature (Coffigniez et al. 2025). Since phytates are known to chelate divalent minerals such as iron, calcium, and zinc, thereby reducing their bioavailability (Chondrou et al. 2024), the observed decrease in heat‐treated flours could contribute to better mineral absorption and improved nutritional quality. The phytate levels obtained in the studied flours were below the daily intake typically provided by diets such as the European or American diet, which can supply up to 2 g of phytate per day (Buades Fuster et al. 2017).

Oxalate content in flours increased with ripening. Pulp flours had higher oxalate levels in *Batard* while whole fruit flours had higher levels in *CARBAP K74*. Oxalate content ranged from 0.07 to 0.31 g/100 g DM and were higher than values reported by Bello et al. (2020) (0.06–0.15 g/100 g DM). The lower oxalate levels observed in precooked flours may be due to the reduction of soluble oxalates during boiling, as cooking in water promotes their leaching (Nha et al. 2022). High intake of soluble oxalate from the diet can increase the risk of kidney stone formation as well as mineral (calcium, iron, magnesium) deficiency (Nha et al. 2022). The Academy of Nutrition and Dietetics has recommended limiting dietary oxalate intake of less than 40–50 mg per day in patients with kidney stones (Avila‐Nava et al. 2021).

Tannins primarily found in banana peels are the main anti‐nutrient in bananas (Emaga et al. 2011). In this study, heat treatments increased tannin levels in *Batard* flours but, in contrast, reduced them in *CARBAP K74* hybrid flours. Tannin content ranged from 0.07 to 0.97 g/100 g DM and was higher than values reported by Ayodele et al. (2019) (0.11 g/100 g DM). Higher levels in whole fruit flours may be due to tannins being more concentrated in the peel than the pulp. According to Yellavila et al. (2015), variations in tannin content can result from factors like variety, ripening, environment, and harvest period. Although tannins can reduce protein digestibility and interfere with iron absorption, their moderate presence (< 1% DM) in the analyzed flour suggests limited adverse nutritional effects. Moreover, tannins also exhibit antioxidant properties that may confer health benefits, effectively scavenging free radicals, reducing oxidative stress, and inhibiting lipid peroxidation (Rudrapal et al. 2022). Overall, the variations observed in phytate, oxalate, and tannin levels indicate that the applied heat treatments, particularly precooking, were effective in reducing anti‐nutrient concentrations to levels compatible with safe consumption and optimal nutrient availability.

### 3.4 Multivariates Analysis From Different Matrices and Treatments Derived From Batard and the Hybrid *CARBAP K74*

**FIGURE 5:**
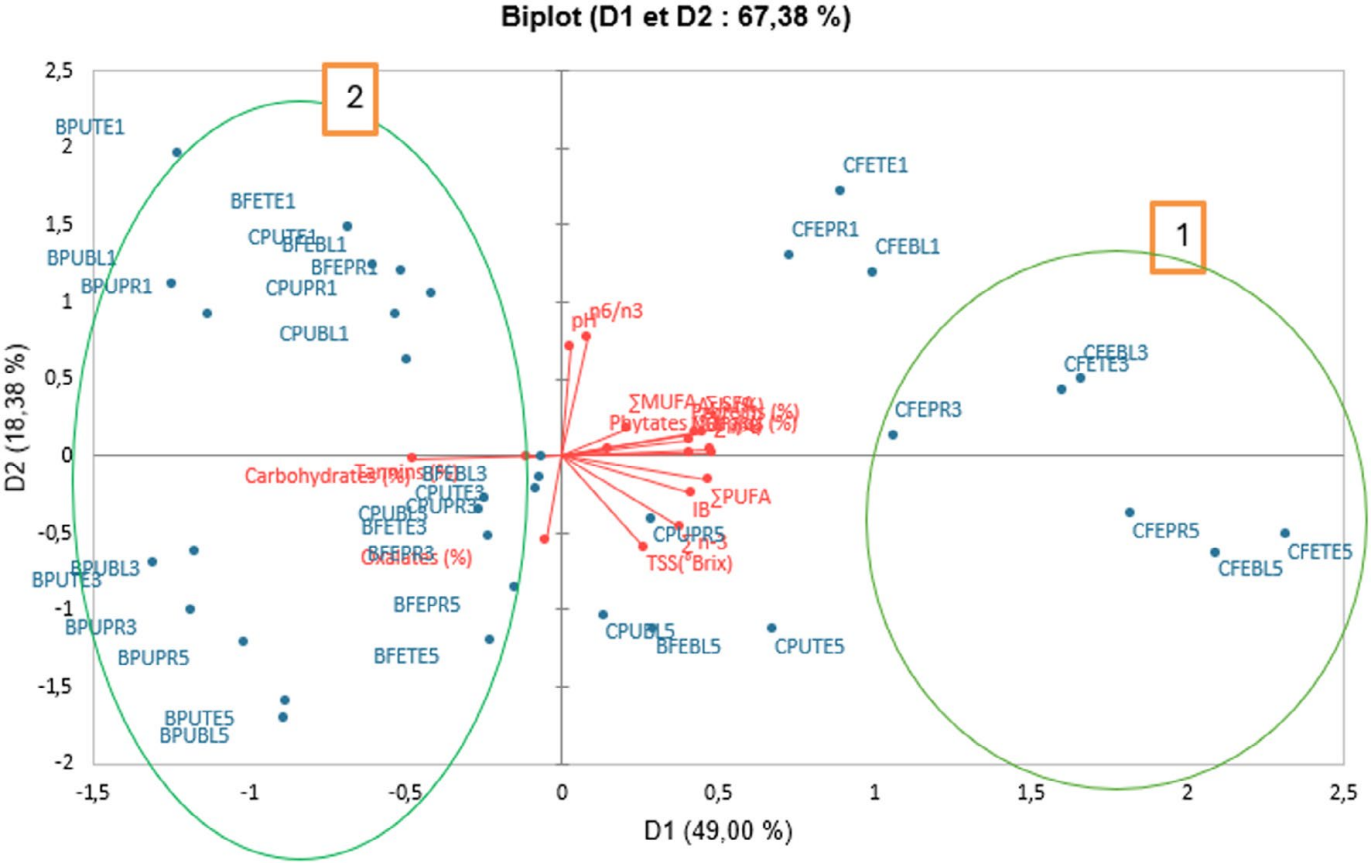
Biplot from Principal Component Analysis (PCA) showing the distribution of active variables (in red) and active observations (in blue) on the first two principal components (D1: 49.00%, D2: 18.38%). (1) Flours characterized by higher soluble solids (TSS), lipid content, and polyunsaturated fatty acids, reflecting improved nutritional quality and potential for functional food applications. (2) Flours characterized by lowest browning index (IB) and reduced levels of antinutritional factors (phytates, tannins, oxalates). **BPUBL1**: *Batard* blanched pulp stage 1, **BPUBL3**: *Batard* blanched pulp stage 3, **BPUBL5**: *Batard* blanched pulp stage 5; **BPUTE1**: *Batard* untreated pulp stage 1; **BPUTE3**: B*atard* untreated pulp stage 3; **BPUTE5**: *Batard* untreated pulp stage 5; **BPUPR1**: *Batard* precooked pulp stage 1; **BPUPR3**: *Batard* Precooked pulp stage 3; **BPUPR5**: *Batard* Precooked pulp stage 5; **BFEBL1**: *Batard* Blanched whole fruit Stage 1; **BFEBL3**: *Batard* blanched whole fruit stage 3; **BFEBL5**: *Batard* blanched whole fruit stage 5; **BFETE1**: *Batard* untreated whole fruit stage 1; **BFETE3**: *Batard* untreated whole fruit stage 3; **BFETE5**: *Batard* untreated whole fruit stage 5; **BFEPR1**: *Batard* Precooked whole fruit stage 1; **BFEPR3**: *Batard* Precooked whole fruit stage 3; **BFEPR5**: *Batard* Precooked whole fruit stage 5; **CPUBL1**: *CARBAP K74* blanched pulp stage 1; **CPUBL3**: *CARBAP k74* blanched pulp stage 3; **CPUBL5**: *CARBAP K74* blanched pulp stage 5; **CPUTE1**: *CARBAP K74* untreated pulp stage 1; **CPUTE3**: *CARBAP K74* untreated pulp stage 3; **CPUTE5**: *CARBAP K74* untreated pulp stage 5; **CPUPR1**: *CARBAP K74* precooked pulp stage 1; **CPUPR3**: *CARBAP K74* Precooked pulp stage 3; **CPUPR5**: *CARBAP K74* precooked pulp stage 5; **CFEBL1**: *CARBAP K74* blanched whole fruit stage 1; **CFEBL3**: *CARBAP K74* blanched whole fruit stage 3; **CFEBL5**: *CARBAP K74* blanched whole fruit stage 5; **CFETE1**: *CARBAP K74* untreated whole fruit stage 1; **CFETE3**: *CARBAP K74* untreated whole fruit stage 3; **CFETE5**: *CARBAP K74* untreated whole fruit stage 5; **CFEPR1**: *CARBAP K74* precooked whole fruit stage 1; **CFEPR3**: *CARBAP K74* precooked whole fruit stage 3; **CFEPR5**: *CARBAP K74* precooked whole fruit stage 5.

**FIGURE 6:**
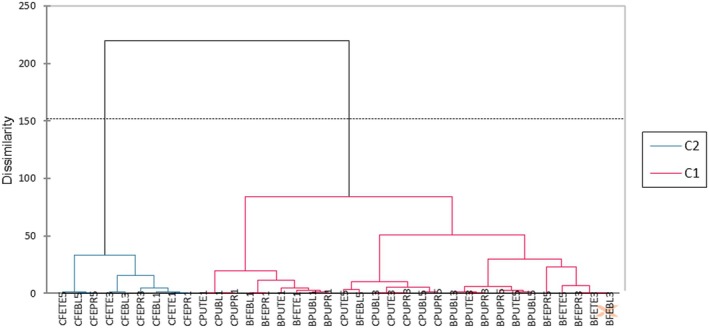
Hierarchical cluster analysis (HCA)/number of clusters = 2. **C1 (BPUBL1**: *Batard* blanched pulp stage 1, **BPUBL3**: *Batard* blanched pulp stage 3, **BPUBL5**: *Batard* blanched pulp stage 5; **BPUTE1**: *Batard* untreated pulp stage 1; **BPUTE3**: *Batard* untreated pulp stage 3; **BPUTE5**: *Batard* untreated pulp stage 5; **BPUPR1**: *Batard* precooked pulp stage 1; **BPUPR3**: *Batard* Precooked pulp stage 3; **BPUPR5**: *Batard* Precooked pulp stage 5; **BFEBL1**: *Batard* Blanched whole fruit Stage 1; **BFEBL3**: *Batard* blanched whole fruit stage 3; **BFEBL5**: *Batard* blanched whole fruit stage 5; **BFETE1**: *Batard* untreated whole fruit stage 1; **BFETE3**: *Batard* untreated whole fruit stage 3; **BFETE5**: *Batard* untreated whole fruit stage 5; **BFEPR1**: *Batard* Precooked whole fruit stage 1; **BFEPR3**: *Batard* Precooked whole fruit stage 3; **BFEPR5**: *Batard* Precooked whole fruit stage 5; **CPUBL1**: *CARBAP K74* blanched pulp stage 1; **CPUBL3**: *CARBAP K74* blanched pulp stage 3; **CPUBL5**: *CARBAP K74* blanched pulp stage 5; **CPUTE1**: *CARBAP K74* untreated pulp stage 1; **CPUTE3**: *CARBAP K74* untreated pulp stage 3; **CPUTE5**: *CARBAP K74* untreated pulp stage 5; **CPUPR1**: *CARBAP K74* precooked pulp stage 1; **CPUPR3**: *CARBAP K74* Precooked pulp stage 3; **CPUPR5**: *CARBAP K74* precooked pulp stage 5; **CFEBL1**: *CARBAP K74* blanched whole fruit stage 1; **CFEBL3**: *CARBAP K74* blanched whole fruit stage 3; **CFEBL5**: *CARBAP K74* blanched whole fruit stage 5). **C2** (**CFETE1**: *CARBAP K74* untreated whole fruit stage 1; **CFETE3**: *CARBAP K74* untreated whole fruit stage 3; **CFETE5**: *CARBAP K74* untreated whole fruit stage 5; **CFEPR1**: *CARBAP K74* precooked whole fruit stage 1; **CFEPR3**: *CARBAP K74* precooked whole fruit stage 3; **CFEPR5**: *CARBAP K74* precooked whole fruit stage 5).

Principal Component Analysis (PCA) and Hierarchical Cluster Analysis (HCA) were performed to assess the relationships among the different flours. The PCA revealed that the first two components explained 67.38% of the total variance, reflecting strong correlations between nutritional variables and physicochemical changes associated with thermal treatments, varieties, and ripening stages (Figure 5). Consistently, the HCA based on Euclidean distance, grouped the flours into two distinct clusters according to their similarities (Figure 6). Axis 1 (D1, 49.00%) is mainly associated with energy and lipid‐related variables (∑*n*−6, ∑PUFA, lipids, carbohydrates, ∑SFA), which are strongly influenced by blanching and precooking treatments. These treatments promote better lipid retention and higher concentrations of polyunsaturated fatty acids, particularly in the *CARBAP K74* variety, which is naturally rich in lipids. Axis 2 (D2, 18.38%) is dominated by the n6/n3 ratio (27.25%), pH (23.27%), and soluble sugars (16.64%), reflecting the impact of ripening stage on acidity, carbohydrate composition, and fatty acid balance. Samples located on the left side of the plot (D1 < 0), including blanched and precooked flours from Batard and *CARBAP K74* varieties derived from the pulp and whole fruits at ripening stages 1, 3, and 5 related to class 1 (C1) in the HCA. This class is characterized by lower nutritional content (proteins, lipids, fibers NDF, ash), monounsaturated fatty acids (MUFA), polyunsaturated fatty acids (PUFA), total soluble solids (TSS), lowest browning index and higher levels of carbohydrates, oxalates and tannins. This distribution indicates a significant reduction in browning as a result of thermal treatments, likely due to the inhibition of enzymatic and non‐enzymatic reactions responsible for color formation, such as polyphenol oxidation. In contrast, samples from the right side (D1 > 0), related to class 2 (C2), particularly the whole‐fruit flours of *CARBAP K74* at advanced ripening stages (stage 3 and 5), is distinguished by flours with elevated nutritional content (proteins, lipids, ash, fibers NDF), monounsaturated fatty acids (MUFA), polyunsaturated fatty acids (PUFA), total soluble solids (TSS), and phytates. Multivariate results demonstrate how combined thermal treatments and ripening stages influence nutritional quality and functional properties of plantain flour, guiding the selection of flours for industrial or nutritional applications.

## 4 Conclusion

This study is one of the first to comprehensively evaluate both pulp and whole fruit flours of *Batard* and *CARBAP K74* bananas across different ripening stages using multivariate analysis. The results showed that heat treatments, especially precooking, positively influenced the quality of flours by improving color, reducing browning, and lowering anti‐nutritional factors. While flours are typically produced from unripe matrix, fruits at ripening stage 3 and 5 can also yield high‐quality flours with higher soluble solids and naturally sweeter flavors. Whole‐fruit flours of the *CARBAP K74* hybrid obtained by precooking can be incorporated into the formulation of infant porridges or bakery products, partially replacing wheat flour. For instance, of breads containing plantain flour which have been well accepted by consumers in Cameroon. Their use could improve several nutritional and technological parameters, notably: a higher dietary fiber content due to the presence of the peel in the flour; better color and starch digestibility resulting from the partial gelatinization induced by precooking; and a sweeter flavor associated with the advanced post‐harvest ripening stage. Despite these promising results, the study was conducted at laboratory scale, and further investigations are needed to evaluate sensory properties, microbiological and techno‐functional performance as well as industrial feasibility. Overall, this work provides novel insights into the production of banana flours from both pulp and whole fruits, highlighting precooking and the use of different ripening stages as key factors for optimizing nutritional and technological quality.

## Author Contributions

**Annie Takam Ngouno:** conceptualization (equal), data curation (equal), formal analysis (equal), methodology (lead), validation (equal), visualization (equal), writing – original draft (lead), writing – review and editing (equal). **Cédric Kendine Vepowo:** conceptualization (equal), data curation (equal), formal analysis (equal), methodology (equal), writing – original draft (equal), writing – review and editing (equal). **Dallonnes Fangueng Kamgo:** conceptualization (equal), data curation (equal), formal analysis (equal), methodology (equal), writing – original draft (equal), writing – review and editing (equal). **Samiha Boutaleb:** conceptualization (equal), data curation (equal), formal analysis (equal), methodology (equal), writing – original draft (equal), writing – review and editing (equal). **Jean‐Luc Hornick:** formal analysis (equal), validation (equal), writing – original draft (equal), writing – review and editing (equal). **Marie‐Louise Scippo:** funding acquisition (equal), supervision (equal), writing – original draft (equal), writing – review and editing (equal). **Gérard Bertin Ngoh Newilah:** methodology (equal), supervision (equal), validation (equal), visualization (equal), writing – original draft (equal), writing – review and editing (equal). **Caroline Douny:** funding acquisition (equal), methodology (equal), supervision (equal), validation (equal), visualization (equal), writing – original draft (equal), writing – review and editing (equal).

## Ethics Statement

The authors have nothing to report.

## Conflicts of Interest

The authors declare no conflicts of interest.

## Data Availability

The data that support the findings of this study are available from the corresponding author upon reasonable request.

## References

[fsn371541-bib-0001] Aina, V. , B. Sambo , A. Zakari , H. Haruna , K. Umar , and R. Akinboboye . 2012. “Determination of Nutritional and Ant Nutritional Content of *Vitis vnifera* (Grapes) Grown in Bomo (Area C) Zaira. Nigeria.” Advance Journal of Food Science and Technology 4, no. 6: 225–228.

[fsn371541-bib-0002] Ajayeoba, T. , O. Kaka , and O. Ajibade . 2021. “7 Microbial Food Spoilage of Selected Food and Food Products.” in Food Science and Technology. Edited by: Oluwatosin Ademola Ijabadeniyi . 10.1515/9783110667462-007.

[fsn371541-bib-0058] Akinjayeju, O. , A. O. Badrudeen , and M. Q. Soretire . 2020. “Comparative Evaluation of Nutritional and Anti‐Nutritional Properties of Peeled, Unpeeled and Blanched Plantain (Musa AAB) Flours and Consumer Acceptability of Their Dumplings.” Asian Food Science Journal 17, no. 2: 10–23.

[fsn371541-bib-0004] AOAC . 1990. Official Methods of Analysis of the Association of Official Analytical Chemists. 15th ed, 1105–1106. Association of Official Analytical Chemists.

[fsn371541-bib-0003] AOAC . 2012. Official Methods of Analysis of AOAC International. 19th ed. Association of Official Analytical Chemists.

[fsn371541-bib-0005] Arshad, M. T. , S. Maqsood , R. Altalhi , et al. 2025. “Role of Dietary Carbohydrates in Cognitive Function: A Review.” Food Science & Nutrition 13, no. 7: e70516. 10.1002/fsn3.70516. 40599356 PMC12209867

[fsn371541-bib-0006] Avila‐Nava, A. , I. Medina‐Vera , P. Rodríguez‐Hernández , et al. 2021. “Oxalate Content and Antioxidant Activity of Different Ethnic Foods.” Journal of Renal Nutrition 31, no. 1: 73–79. 10.1053/j.jrn.2020.04.006. 32709427

[fsn371541-bib-0057] Ayodele, A. , F. Imoleayo , and A. Adeniyi . 2019. “The Effect of Processing Method on the Proximate, Anti‐Nutrient and Phytochemical Composition of Ripe and Unripe Plantain (*Musa paradisiaca*).” Open Science Journal of Analytical Chemistry 4, no. 1: 1–6.

[fsn371541-bib-0040] Bancal, V. , and R. Ray . 2024. “Overview of Food Loss and Waste in Fruits and Vegetables: From Issue to Resources.” In Fruits and Vegetable Wastes, 1. Springer. 10.1007/978-981-16-9527-8_1.

[fsn371541-bib-0056] Bello, F. A. , E. M. Akpan , and M. A. Sodipo . 2020. “Physicochemical and Sensory Properties of Cookies Produced from Wheat, Unripe Plantain and Germinated Fluted Pumpkin Seed Composite Flour.” Food Science and Quality Management 96: 36–43.

[fsn371541-bib-0007] Blasco, L. G. , and F. Montano . 2015. “Propiedades Funcionales Del Platano (Musasp).” Revista Medica de la Universidad Veracruzana 14, no. 2: 22–26.

[fsn371541-bib-0009] Buades Fuster, J. M. , P. Sanchís Cortés , J. Perelló Bestard , and F. Grases Freixedas . 2017. “Plant Phosphates, Phytate and Pathological Calcifications in Chronic Kidney Disease.” Nefrología 37: 20–28. 27697413 10.1016/j.nefro.2016.07.001

[fsn371541-bib-0049] Campuzano, A. , C. M. Rosell , and F. Cornejo . 2018. “Physicochemical and Nutritional Characteristics of Banana Flour During Ripening.” Food Chemistry 256: 11–17. 29606425 10.1016/j.foodchem.2018.02.113

[fsn371541-bib-0010] Chondrou, T. , N. Adamidi , D. Lygouras , S. A. Hirota , O. Androutsos , and V. Svolos . 2024. “Dietary Phytic Acid, Dephytinization, and Phytase Supplementation Alter Trace Element Bioavailability A Narrative Review of Human Interventions.” Nutrients 16, no. 23: 4069. 10.3390/nu16234069. 39683463 PMC11643945

[fsn371541-bib-0011] CODEX . 1985. “Standard for Wheat Flour.”

[fsn371541-bib-0012] Coffigniez, F. , R. Melina , and V. Lullien‐Pellerin . 2025. “Phytic Acid Decrease Along Hydrothermal Treatment of Bambara Groundnuts: A Deep Assessment of the Mechanisms Involved.” LWT 228: 118133. 10.1016/j.lwt.2025.118133.

[fsn371541-bib-0013] Dadzie, B. , and J. Orchard . 1997. Routine Post‐Harvest Screening of Banana/Plantain Hybrids: Criteria and Methods, 75. INIBAP Technical Guidelines. INIBAP.

[fsn371541-bib-0014] Donlao, N. , B. Nasuha , and P. Asia . 2020. “Utilization of Banana Agricultural Waste ‐ Effects of Processing Conditions on Properties of Unripe Banana (*Musa cavendish*) Pulp and Peel Flours.” Engineering in Agriculture, Environment and Food 13, no. 4: 129–138. 10.37221/eaef.13.4_129.

[fsn371541-bib-0015] Douny, C. , R. El Khoury , J. Delmelle , et al. 2015. “Effect of Storage and Cooking on the Fatty Acids Profile of Omega‐3 Enriched Eggs and Pork Meat Marketed in Belgium.” Food Science & Nutrition 3: 140–152. 10.1002/fsn3.197. 25838892 PMC4376408

[fsn371541-bib-0050] DREES . 2017. “Direction de la recherche, des études, de l'évaluation et des statistiques.” L'état de santé de la Population en France, Rapport 2017.

[fsn371541-bib-0016] Dziki, D. 2023. “The Latest Innovations in Wheat Flour Milling: A Review.” Agricultural Engineering 27: 147–162. 10.2478/agriceng-2023-0011.

[fsn371541-bib-0017] Emaga, H. , C. Robert , N. Ronkart , B. Wathelet , and M. Paquot . 2011. “Dietary Fiber Components and Pectin Chemical Features of Peels During Ripening in Banana and Plantain Varieties.” Bioresource Technology 99, no. 4346: 4354. 10.1016/j.biortech.2007.08.030. 17931857

[fsn371541-bib-0018] Fadimu, G. , and L. Oladimeji . 2018. “Effect of Drying Methods on the Chemical Composition. Colour. Functional and Pasting Properties of Plantain (Musa Parasidiaca) Flour.” Croatian Journal of Food Technology, Biotechnologyand Nutrition 13, no. 1–2: 38–43. 10.31895/hcptbn.13.1-2.2.

[fsn371541-bib-0019] Falk, A. 2018. “Marché de la Banana Plantain.” Fruit Tropical 1, no. 256: 50–55.

[fsn371541-bib-0021] FAO . 2024. “FAOSTAT Statistical Database.” Food and Agriculture Organization of the United Nations. https://www.fao.org/faostat/en/#home.

[fsn371541-bib-0053] Fidrianny, I. , S. Anggraeni , and M. Insanu . 2018. “Antioxidant Properties of Peels Extracts From Three Varieties of Banana (*Musa* sp.) Grown in West Java, Indonesia.” International Food Research Journal 25: 57–64.

[fsn371541-bib-0022] Fofiri, E. , and L. Temple . 2023. “Politique D'import‐Substitution au blé et Compétitivité des Farines Panifiables à Base de Manioc, Banane Plantain et Patate Douce au Cameroun.”

[fsn371541-bib-0023] Folch, J. , M. Lees , and G. Stanley . 1957. “A Simple Method for the Isolation and Purification of Total Lipids From Animal Tissues.” Journal of Biological Chemistry 226: 497–509. 10.1016/S0021-9258(18)64849-5. 13428781

[fsn371541-bib-0024] Guessan, A. , K. Olivier , and J. Gonnety . 2018. “Effect of Chemical and Thermal Treatments on Browning Inhibition of Senescent Plantain (Musa Paradisiaca) Puree for Semolinas Preparation.” American Journal of Biochemistry 8, no. 4: 75–84. 10.5923/j.ajb.20180804.02.

[fsn371541-bib-0025] Gupta, S. , M. Sood , N. Gupta , J. Bandral , and A. Langeh . 2022. “Food Browning, Its Type and Controlling Measures: A Review Article.” Chemical Science Review and Letters 11, no. 1: 29–45. 10.37273/chesci.cs205210390.

[fsn371541-bib-0026] Hunt, G. 1991. Measuring Colour. 2nd ed, 75–76. Ellis Horwood.

[fsn371541-bib-0055] Iliyasu, R. , H. Ayo Omogie , H. H. Adamu , and A. Adamu . 2019. “Effect of Ripening and Pretreatment on the Physical, Pasting, and Sensory Properties of Cardaba Banana (Musa ABB) Flour.” Bayero Journal of Pure and Applied Sciences 12: 651–656.

[fsn371541-bib-0027] ISO . 2006. “Aliments Des Animaux.” Détermination du contenu en fibre par traitement à l'amylase et au détergent neutre (aNDF).

[fsn371541-bib-0028] Jimenez Moreno, J. A. , T. Linhares Cruz Tabosa Barroso , L. E. Nochi Castro , et al. 2025. “Unlocking the Industrial Potential of Cambuci Peel: A Sustainable Approach Based on Its Physicochemical Profile.” Resources 14, no. 7: 109. 10.3390/resources14070109.

[fsn371541-bib-0029] Kaewjumpol, G. , S. Srisamlee , D. M. Beckles , and K. Luengwilai . 2021. “Enzymatic Browning in Banana Blossoms and Techniques for Its Reduction.” Horticulturae 7, no. 10: 373. 10.3390/horticulturae7100373.

[fsn371541-bib-0030] Kiin‐Kabari, D. 2020. “The Effect of Chemical Treatments on the Browning Prevention of Plantain (*Musa paradisiaca*) Products.” Journal of Food Stability 3, no. 2: 1–8. 10.36400/J.FOOD.STAB.3.2.2020-0011.

[fsn371541-bib-0031] Kim, Y. , M. Singh , and S. Kays . 2007. “Near‐Infrared Spectroscopic Analysis of Macronutrients and Energy in Homogenized Meals.” Food Chemistry 105: 1248–1255. 10.1016/j.foodchem.2007.03.011.

[fsn371541-bib-0051] Kumar, S. , A. Saravanan , N. Sheeba , and S. Uma . 2019. “Structural, Functional Characterization and Physicochemical Properties of Green Banana Flour From Dessert and Plantain Bananas (*Musa* spp.).” Plant Foods for Human Nutrition 116: 108524.

[fsn371541-bib-0032] Loranger‐Merciris, G. , G. Damour , B. Deloné‐Louis , et al. 2023. “Management Practices and Incidence of Pests in Plantain (Musa Paradisiaca AAB) Crops. Consequences on the Sustainability of the Cropping Systems.” Applied Soil Ecology 189: 104904. 10.1016/j.apsoil.2023.104904.

[fsn371541-bib-0033] Luque, V. , P. Crespo‐Escobar , E. M. Hård af Segerstad , et al. 2024. “Gluten‐Free Diet for Pediatric Patients With Coeliac Disease: A Position Paper From the ESPGHAN Gastroenterology Committee, Special Interest Group in Coeliac Disease.” Journal of Pediatric Gastroenterology and Nutrition 78, no. 4: 973–995. 10.1002/jpn3.12079. 38291739

[fsn371541-bib-0034] Mundéné‐Timothée, J. , A. Nouga Bissoue , R. M. Nguimbou , et al. 2024. “Plantain Flour: Production Processes, Technological Characteristics, and Its Potential Use in Traditional African Dishes–A Review.” Journal of the Science of Food and Agriculture 105: 4741–4752. 10.1002/jsfa.13900. 39360957

[fsn371541-bib-0035] Ndhlala, A. R. , A. Kasiyamhuru , C. Mupure , K. Chitindingu , M. A. Benhura , and M. Muchuweti . 2007. “Phenolic Composition of *Flacourtia indica* , Opuntia Megacantha and *Sclerocarya birrea* .” Food Chemistry 103, no. 1: 82–87. 10.1016/j.foodchem.2006.06.066.

[fsn371541-bib-0036] Ngoh, G. , T. Nafack , T. Tembe , M. Nkouandou , E. Ngombi , and C. Asseng . 2017. “Pancake Formulations Based on Plantain Flour (*Musa AAB*).” International Journal of Food Science and Biotechnology 2, no. 3: 87–96. 10.11648/j.ijfsb.20170203.13.

[fsn371541-bib-0037] Ngoh Newilah, G. , C. Kendine Vepowo , R. Nya Nzimi , et al. 2024. “User Preferences and Consumer Acceptability of Boiled Plantain in Rural and Urban Localities in Cameroon.” Journal of the Science of Food and Agriculture 104, no. 8: 4838–4850. 37910398 10.1002/jsfa.13090

[fsn371541-bib-0038] Nha, K. , D. H. Huynh , H. Nguyen , and V. Nguyen . 2022. “Effects of Processing on Oxalate Contents in Plant Foods.” A Review, Journal of Food Composition and Analysis 112: 104685, ISSN 0889‐1575. 10.1016/j.jfca.2022.104685.

[fsn371541-bib-0039] Pathare, P. , U. Opara , and F. Al‐Said . 2013. “Colour Measurement and Analysis in Fresh and Processed Foods: A Review.” Food and Bioprocess Technology 6: 36–60. 10.1007/s11947-012-0867-9. PMC7089433 32215166

[fsn371541-bib-0059] Rudrapal, M. , P. Khairnar , J. Khan , et al. 2022. “Dietary Polyphenols and Their Role in Oxidative Stress and Human Health: A Review.” Frontiers in Pharmacology 13: 806470. 35237163 10.3389/fphar.2022.806470 PMC8882865

[fsn371541-bib-0041] Salazar, D. , M. Arancibia , D. Lalaleo , R. Rodríguez‐Maecker , M. E. López‐Caballero , and M. Montero . 2022. “Physico‐Chemical Properties and Filmogenic Aptitude for Edible Packaging of Ecuadorian Discard Green Banana Flours (Musa Acuminanta AAA).” Food Hydrocolloids 122: 107048. 10.1016/j.foodhyd.2021.107048.

[fsn371541-bib-0052] Setiarto, R. , T. Fitrilia , S. Masyrifah , and L. Amalia . 2020. “Effect of Blanching on the Physicochemical Characteristics and Microstructure of Canistel Seed Flour (*Pouteria campechiana* (Kunth) Baehni).” African Journal of Food Agriculture Nutrition and Development 20: 17063–17080.

[fsn371541-bib-0042] Singh, R. , S. Ranvir , and S. Madan . 2017. “Comparative Study of the Properties of Ripe Banana Flour, Unripe Banana Flour and Cooked Banana Flour Aiming Towards Effective Utilization of These Flours.” International Journal of Current Microbiology and Applied Sciences 6, no. 8: 2003–2015. 10.20546/ijcmas.2017.603.239.

[fsn371541-bib-0043] Soto‐Maldonado, C. , J. Concha‐Olmos , and M. Zúñiga‐Hansen . 2020. “The Effect of Enzymatically Treated Ripe Banana Flour on the Sensory Quality and Glycemic Response of Banana‐Wheat Flour Composite Muffins.” Journal of Food Science and Technology 57, no. 10: 3621–3627. 10.1007/s13197-020-04394-6. 32903937 PMC7447685

[fsn371541-bib-0044] Takahashi, M. , J. Ando , K. Shimada , et al. 2017. “The Ratio of Serum n‐3 to n‐6 Polyunsaturated Fatty Acids Is Associated With Diabetes Mellitus in Patients With Prior Myocardial Infarction: A Multicenter Cross‐Sectional Study.” BMC Cardiovascular Disorders 17: 1–10. 10.1186/s12872-017-0479-4. 28125968 PMC5270364

[fsn371541-bib-0060] Udomkun, P. , C. Masso , R. Swennen , et al. 2021. “How Does Cultivar, Maturation, and Pre‐Treatment Affect Nutritional, Physicochemical, and Pasting Properties of Plantain Flours?” Food 10: 1749. 10.3390/foods10081749 PMC8393996 34441527

[fsn371541-bib-0045] Virulchatapan, P. , and N. Luansakum . 2020. “Effet of Harvesting Periods on Physiochemical Properties and in Vitro Digestibility of Banana Flour.” International Journal of Agricultural Technology 16, no. 2: 517–528.

[fsn371541-bib-0046] von Braun, J. , B. B. Chichaibelu , D. Laborde Debucquet , and M. Torero . 2024. “Cost of Ending Hunger: Consequences of Complacency, and Financial Needs for SDG2 Achievement (No. 347).” ZEF Discussion Papers on Development Policy.

[fsn371541-bib-0047] Wani, K. , and M. Dhanya . 2025. “Unlocking the Potential of Banana Peel Bioactives: Extraction Methods, Benefits, and Industrial Applications.” Discover Food 5, no. 8. 10.1007/s44187-025-00276-y.

[fsn371541-bib-0048] Yellavila, S. , J. Agbenorhevi , J. Asibuo , and G. Sampson . 2015. “Proximate Composition, Minerals Content and Functional Properties of Five Lima Bean Accessions.” Journal of Food Security 3, no. 3: 69–74. 10.12691/jfs-3-3-1.

